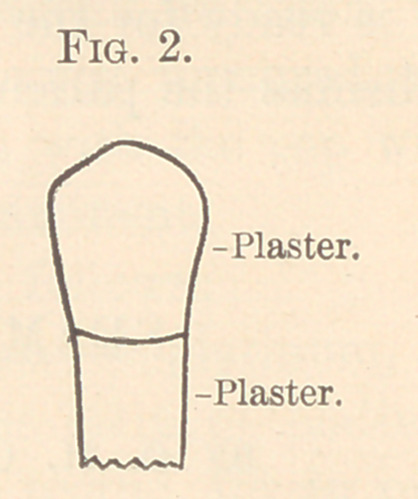# Seamless Gold Crowns

**Published:** 1903-10

**Authors:** C. M. Glazier

**Affiliations:** Boston, Mass.


### SEAMLESS GOLD CROWNS.

BY C. M. GLAZIER, D.M.D., BOSTON, MASS.

Some of us have wanted to make seamless gold crowns, have bought some system, and failed because we depended upon the machine to make the crown. The machine will produce a crown, but to make a crown that will fit with perfect articulation the machine should only be used to assist.

One important point is the production of a model, and that may be accomplished by the following method.

Make some seamless bands of copper 32 gauge of various sizes. This can be done by drawing a cartridge with a draw-plate and cutting off the top by driving into a smaller hole of the draw-plate. Select a band that will fit the prepared root, festooning and placing it below the gum margin the desired distance. Take impression and bite. Placing band in impression, pour model and place on articulator. Now fill the band with plaster and close the articulator, having previously varnished the models. When the plaster has set, carve to tooth form. (Fig. 1.)

If it is a broken tooth or root you are crowning, you can swage into a counter-die; if it is a perfect tooth prepared as an abutment, swage over a die.

To swage into a counter-die remove the model of the tooth from the articulator, fill the bottom of the band with mouldine, insert a tooth-pick, and push into fusible metal poured into a Berry swager. Split the metal to remove the model. Place a cartridge the size of a band used in the counter-die and swage with cornmeal. The line of demarkation between the mouldine and the band of the model must be made distinct, so as to know where to cut off the crown.

To swage over a die, space must be left between the model of the tooth and the occluding tooth the thickness of the gold to be used. Then cut the model of the tooth from the articulator, letting the plaster extend from the bottom of the band; then remove the band and carve as in Fig. 2. The line of demarkation must

Fig. 1.

Fig. 2.

be made distinct between the crown model and extending plaster. Then pour soft plaster into two half-circles of zinc one inch high, placed together and held by an elastic band, and insert model of tooth. When the plaster is dry fracture through the centre and remove the model, place together again, and pour fusible metal into the counter-die to produce the metal die.

Place a cartridge of gold over the die and with swaging hammer draw to fit the model, placing lastly in swager with heavy plunger, using mouldine to swage with. When swaged to fit, boil out fusible metal, trim, and polish the crown.

To acquire the right direction of force the two apposing surfaces of the swager should be concave.

In the case of a perfect tooth prepared for an abutment, a plaster model of that prepared tooth may be reproduced in metal, and a crown made to fit that model. This is a case where a die method is demanded.

The face of a crown may be cut out and the adaptability of the gold will be found perfect, which is essential to an open-faced crown.

By the adoption of both methods of swaging practicable seamless gold crowns can be made having perfect contours.

## Figures and Tables

**Fig. 1. f1:**
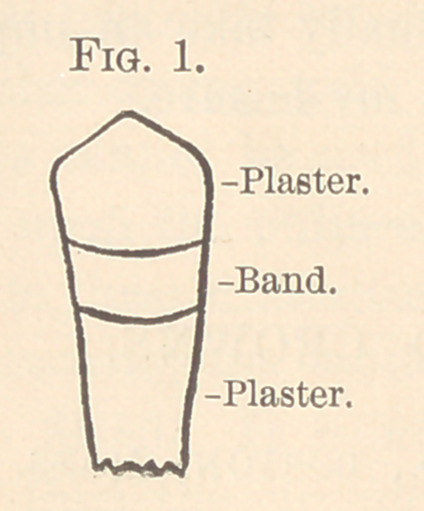


**Fig. 2. f2:**